# Sex-related disparities in hyperlipidaemia and cardiovascular risk in Sri Lanka: a cross-sectional study

**DOI:** 10.1186/s12944-025-02812-2

**Published:** 2025-12-09

**Authors:** Chamila Mettananda, Kavindya Fernando, Maheeka Solangaarachchige, Ravini Premaratna, Randula Mallawa, Upeksha Fernando, Arundika Senaratne, Udara Perera, Champika Wickramasinghe

**Affiliations:** 1 Department of Pharmacology, Faculty of Medicine, University of Kelaniya, Ragama, Sri Lanka; 2 Department of Biochemistry and Clinical Chemistry, Faculty of Medicine, University of Kelaniya, Kelaniya, Sri Lanka; 3 Department of Chemical Pathology, Medical Research Institute, Colombo, Sri Lanka; 4 Examination Unit, Faculty of Medicine, University of Kelaniya, Ragama, Sri Lanka; 5 NCD Directorate, Ministry of Health, Colombo, Sri Lanka

**Keywords:** Sex factors, Cholesterol, Cardiovascular diseases, Hyperlipidaemia, Sri lanka, Health resources

## Abstract

**Background:**

Nearly 80% of the world’s noncommunicable disease (NCD) burden comes from developing countries, where sex-related disparities in healthcare utilisation are common. However, studies on sex-related disparities in lipids and cardiovascular risk among South Asians are limited. This study aimed to investigate sex-related differences in the prevalence, management and outcome of lipids and related cardiovascular diseases (CVDs) among Sri Lankans to inform necessary improvements in preventing, diagnosing, and managing CVDs in females in Sri Lanka, a resource-limited South Asian setting.

**Methods:**

Secondary data from the World Health Organisation’s STEPS survey-2021, a community-based cross-sectional study conducted across Sri Lanka from April to December 2021, were analysed. Hyperlipidaemia was defined as having a total cholesterol (TC) level ≥ 200 mg/dL or being on treatment for hyperlipidaemia. TC goal was defined as a TC level < 200 mg/dL. Rates of hyperlipidaemia prevalence, treatment uptake and TC target achievement were compared between males and females. Sex-related disparities in the associations of hyperlipidaemia and CVDs were assessed separately.

**Results:**

The study sample consisted of 4,634 adults (62.8% female) with a mean age of 46 ± 13.7 years. The prevalence of hyperlipidaemia was higher in females (46.3%) than in males (36.4%, *P* < 0.001). Treatment uptake was not significantly different between females (25.2%) and males (23.6%, *P* = 0.467). Of those on treatment for hyperlipidaemia, the TC goal achievement was low in females (68.1%) compared to males (82.4%, *P* < 0.001). Females (4.9%) had a lower prevalence of established CVDs compared to males (10.9%, *P* < 0.001), but females with hyperlipidaemia had higher odds (OR 1.24, *P* = 0.038) of CVDs.

**Conclusions:**

Sri Lankan females had a higher prevalence and poorer control of hyperlipidaemia compared to males, despite no significant difference in treatment uptake. However, the presence of CVDs was higher among males and was independently associated with sex and hyperlipidaemia.

**Supplementary Information:**

The online version contains supplementary material available at 10.1186/s12944-025-02812-2.

## Background

Cardiovascular diseases (CVDs) are the leading cause of death worldwide, with 80% of cases occurring in low- and middle-income countries [1]. Mortality due to CVDs is rising globally, and this trend is partly driven by the growing population of middle-aged females worldwide [2]. Sex-related disparities in lipid profiles and cardiovascular (CV) risks are well-recognised [2–4]. They are mediated mainly by age and hormonal factors, particularly hormones related to menstruation, pregnancy, breastfeeding, and menopause [2]. Additionally, sex-specific factors unique to females, such as hypertensive disorders of pregnancy, primary ovarian insufficiency, and polycystic ovary syndrome, also contribute to sex-related disparities in lipids as well as CVDs [2, 5]. Autoimmune diseases, which have a greater predilection to affect females, further exacerbate CV risk [6, 7]. These biological vulnerabilities are compounded by sociocultural factors related to sex as well [8–10]. Females and older adults are more likely to discontinue statin therapy [11–13]. Initially, untreated females are less likely than males to initiate statin treatment, regardless of their low-density lipoprotein cholesterol (LDL-C) levels [14, 15]. In addition, the higher prevalence of psychosocial stress, depression, and anxiety [16] among females, along with limited education and awareness of CV risk factors [17, 18], contributes to suboptimal lipid profiles, high CV risk profiles and adverse health outcomes, particularly in developing countries [10, 19–21].

Literature on sex-related disparities in lipid profiles shows evidence that girls, compared to boys, have higher LDL-C and total cholesterol (TC) levels but similar high-density lipoprotein cholesterol (HDL-C) levels [22]. Typically, females have higher TC and HDL-C levels and lower TC/HDL ratios than males [23]. In early life, up to 18–19 years of age, girls have higher LDL-C and TC levels than boys, but HDL-C levels are comparable. From early adulthood to middle age (from 20 to 24 to 55–59 years), females have lower LDL-C and higher HDL-C levels, while LDL-C levels increase and HDL-C levels decrease in males [23]. With older age, all lipid levels decline in both sexes, with a more marked reduction in males [24].

Sex-related disparities in the management and outcome of lipid-related diseases in South Asians, including Sri Lankans, are not fully understood and show discordant results from the global picture. South Asians tend to have lower LDL-C levels compared to Caucasians at any given age [25]. Previously reported literature reveals Sri Lankan females to have higher TC, HDL-C, LDL-C, and triglyceride (TG) values than males across all age groups [26]. A community-based study did not identify sex as being associated with differences in the prevalence of hyperlipidaemia in Sri Lanka [27]. Identifying sex-related disparities in South Asians is especially important as one-third of the world’s population resides in this part of the world. Identifying sex-related disparities helps in the targeted implementation of care in the primary prevention of hyperlipidaemia as well as both primary and secondary prevention of CVDs in females and males.

Therefore, this study aimed to investigate sex-related differences in the prevalence, management and goal achievement of hyperlipidaemia and the sex related differences in the association of hyperlipidaemia and CVDs (stroke, myocardial infarctions and peripheral vascular diseases) in Sri Lankans, which could give insights into South Asian perspectives. The study also aimed to investigate sex-related differences in lipids between urban, rural and estate dwellers. Using nationally representative data, this study will bring context-specific insights that could inform more equitable, sex-sensitive CVD prevention strategies in the region.

## Methodology

Secondary data were analysed from the STEPS Noncommunicable Disease Risk Factor Survey – Sri Lanka 2021, which is part of the World Health Organization’s (WHO) STEPwise approach to noncommunicable disease (NCD) risk factor surveillance (STEPS) under the Adult Risk Factor Surveillance project [28, 29]. This cross-sectional community-based study assessed the prevalence of behavioural and biological risk factors for noncommunicable diseases in Sri Lanka from April to December 2021. Non-Sri Lankans living temporarily and individuals mentally or physically unfit to be included in the study were excluded from the survey. A total of 6267 nationally representative adults, aged 18 to 69, who had lived in their current residence for more than 6 months at the time of sampling, were studied using multi-stage stratified cluster sampling. The sample was stratified based on district and area (urban, rural, estate) levels. A primary sampling unit (PSU) was considered a cluster. The frame of PSUs was based on the census blocks prepared at the Census of Population and Housing – 2011, which was updated for the first quarter Labor Force Survey in 2018. Thus, 644 PSUs were selected using Probability Proportionate Sampling (PPS) method, based on the population of the area [29] The study sample was selected using population data from the Department of Census and Statistics (DCS). Data was collected in three steps: an interviewer administered questionnaire, physical measurements, and biochemical measurements. The methodology was published previously [28]. Step 1 involved collecting data on sociodemographic characteristics, behavioural risk factors for NCDs and factors related to an increase in the physiological risk using an interviewer-administered questionnaire. Step 2 included collecting physical measurements such as height, weight, waist circumference, blood pressure and spot urine collection from the study participants. Finally, step 3 involved collecting biochemical measurements to assess fasting plasma glucose and serum TC levels.

De-identified survey data (*N* = 6267) were extracted, and only participants with complete TC data (*N* = 4634) were included in the analysis (Supplementary Table 1). Hyperlipidaemia was defined according to the Adult Treatment Panel III (ATP III) guidelines by the National Cholesterol Education Programme (NCEP) as having a TC ≥ 200 mg/dL or currently being on treatment for hyperlipidaemia [30]. Sex was defined as a set of biological features associated with physical and physiological features, including chromosomes, gene expression, hormone function and reproductive/sexual anatomy [31]. Level of education was determined by the total number of completed years of schooling (excluding pre-school). Participants were then grouped into six mutually exclusive categories based on the highest qualification attained: (1) no formal schooling, (2) up to Grade 5, (3) passed Grades 6–10, (4) passed G.C.E. ordinary level (O/L), (5) passed G.C.E. advanced level (A/L), (6) degree or higher level of education. Main occupation categories followed the national STEPS coding as: “engaged in economic activity” (any paid employment or self-employment), “seeking and available for work” (unemployed and actively seeking work), “student”, “engaged in household activities” (full-time unpaid domestic work/caregiving),“retired”, “unable to work (elderly/disabled)”, and “other” [28, 29].

Body mass index (BMI) was calculated by dividing an individual’s weight in kilograms by the square of their height in meters. The average of the last two out of three systolic blood pressure (SBP) readings was used as the current SBP. The predicted 10-year CV risk was calculated in participants aged 40 years or above, without a history of CVDs, and with complete data for CV risk calculation (*N* = 2667). The wealth index was calculated according to the method published before, and patients were categorised into five wealth quintiles (from 1 (poorest) to 5 (richest) [29]. Ten-year CV risk in females and males of 40 to 79 years of age without existing CVDs were calculated using the 2019 WHO CV risk prediction charts for the South-East Asia Region (SEAR) using the R statistical package [32, 33]. These charts estimate 10-year CV risk based on age, sex, smoking status, diabetes status, SBP, and TC levels. A high CV risk was defined as a 10-year risk of 20% or more [34]. The lifetime CV risk in females and males of 20 to 59 years of age without existing CVDs was calculated using the ASCVD Risk Estimator (Available at: https://tools.acc.org/ldl/ascvd_risk_estimator) and a Python-based program [1]. Patients were stratified into two risk categories: low- (< 39%), and high-lifetime risk (≥ 39%), following the American College of Cardiology (ACC) and American Heart Association (AHA) guidelines [35, 36].

The prevalence estimates of hyperlipidaemia, treatment uptake rates and the percentage of individuals with normal TC level (TC < 200 mg/dL) were stratified according to sector of residence and compared across males and females. TC treatment goal achievement was defined as having TC < 200 mg/dL in individuals receiving treatment for hyperlipidaemia. Continuous variables were assessed for normality and expressed as means with standard deviations (SD) if normally distributed and medians with interquartile ranges (IQR) if not. Differences between groups were assessed using the independent samples t-test for normally distributed variables and the Mann–Whitney U test for non-normally distributed variables. Categorical variables were expressed as frequencies and percentages, with group comparisons performed using the chi-square (χ2) test. Stratified analyses were performed by age group (< 50 years and ≥ 50 years) to find associations related to the pre-and post-menopausal status, based on the average age of menopause in Sri Lanka (50th centile 50.18, 95% CI 49.51.99 years) [37, 38]. Binary logistic regression analysis was conducted to identify factors associated with hyperlipidaemia, achievement of the TC target, and the presence of existing CVDs. Existing CVDs were captured via self-report using standard WHO STEPS questions, ever having a heart attack, chest pain from heart disease (angina), or a stroke. All statistical tests were two-sided; a *P*-value ≤ 0.05 was considered statistically significant. Analysis was conducted using IBM SPSS Statistics for Windows, Version 20.

All procedures performed in this study adhered to the Helsinki Declaration’s ethical principles and professional conduct standards. Ethics approval for the STEPS study in Sri Lanka was obtained from the Ethics Review Committee of the Sri Lanka Medical Association (ERC/17–021).

## Results

A total of 4634 participants between 18 and 69 years were included in the analysis. Of them, 2914(62.8%) were females and 1720(37.3%) were men, with mean ages of 45.5 ± 13.5 years and 46.4 ± 13.9 years respectively. Of the female participants, 38 (1.3%) were pregnant mothers. The baseline characteristics of the study population are presented in Table 1. Overall, females were younger (*P* = 0.015) and had lower rates of smoking (*P* < 0.001), alcohol consumption (*P* < 0.001), stroke (*P* = 0.015), ischaemic heart disease (*P =* 0.014) and had lower SBP (*P* < 0.001). Average years of schooling were more in females (9.9 years, SD 3.5) than in males (9.7 years, SD 3.5) (*P* = 0.039). The mean waist circumference and BMI were significantly higher in females compared to males; 87.4 (SD 87.4) vs. 86.2 (SD 12.2) cm, *p* = 0.003 and 25.2(SD 5.5) vs. 23.3(SD 4.6) kg/m^2,^ *p* < 0.00, respectively. However, the mean systolic blood pressure in females was lower than in males, 127.9 (SD 22.2) vs. 131 (SD 20.3) mmHg, < 0.001. There was no difference in the fasting blood glucose level between females and males, 106.3(SD 64.2) vs. 106.3(46.9), *p* = 0.853.

**Table 1:** Baseline characteristics of the study population

	Females *N*(%) *N* = 2914	Males *N*(%) *N* = 1720	*P* value^a^
Age groups			0.002
18–29	418 (14.3)	249 (14.5)	
30–44	1015 (34.8)	507 (29.4)	
45–59	893 (30.6)	588 (34.2)	
60–69	588 (20.2)	378 (21.9)	
Age ≥ 50 years	1183(40.6)	767(44.6)	0.008
Level of education			0.003
No schooling	102 (3.5)	40 (2.3)	
Up to grade 5	350 (12.0)	244 (14.2)	
Passed grade 6–10	1285 (44.1)	823 (47.8)	
Passed G.C.E. O/L	502 (17.2)	266 (15.5)	
Passed G.C.E. A/L	536 (18.4)	266 (15.5)	
Degree and above	138 (4.7)	80 (4.7)	
Level of education	1 (0.0)	1 (0.1)	
Main Occupation			< 0.001
Engaged in economic activity	905 (31.1)	1411 (82.0)	
Seeking for and available work	41 (1.4)	49 (2.8)	
Student	110 (3.8)	72 (1.1)	
Engaged in household activities	1723 (59.1)	14 (0.8)	
Retired	58 (2.0)	110 (6.4)	
Unable to work (elderly/disable)	75 (2.6)	61 (3.5)	
Other	2 (0.1)	3 (0.2)	
Income category (by monthly household income in LKR), n (%) (N=*2905, **1713)			< 0.001
*Category 1* (≤ 10 000)	214 (7.4)	76 (4.4.)	
*Category 2* (10 001–23 500)	478 (16.6)	265 (15.5)	
*Category 3* (23 501–36 500)	689 (23.7)	420 (24.5)	
*Category 4* (36 501–52 000)	752 (25.9)	470 (27.4)	
*Category 5 (*52 001–81 500)	471 (16.2)	270 (15.8)	
*Category 6 (*> 81 501)	288 (9.9)	208 (12.1)	
Do not know	13 (0.3)	4 (0.1)	
Wealth index			0.255
Poorest quintile	606 (20.8)	322 (18.7)	
Second quintile	573 (19.7)	346 (20.1)	
Third quintile	614 (21.1)	349 (20.3)	
Fourth quintile	580 (19.9)	347 (20.2)	
Richest quintile	541 (18.6)	356 (20.2)	
Sector of residence			0.170
Urban	417 (14.3)	272 (15.8)	
Rural	2396 (82.2)	1377 (80.1)	
Estate	101 (3.5)	71 (4.1)	
Behavioural Risk factors			
Current smokers	3 (0.1)	502 (29.2)	< 0.001
Alcohol consumption within the last 7days	8 (0.3)	473 (27.5)	< 0.001
Inadequate vegetable/fruit consumption (N=*2881, **1695)	1421 (49.3)	860 (50.7)	0.355
Inadequate physical activity (N=*207, **241)	98 (47.3)	104(43.2)	0.374
Medical history			
Hypertension	753 (25.8)	479 (27.8)	0.135
Diabetes mellitus	479 (16.4)	289 (16.8)	0.747
Stroke	21 (0.7)	25 (1.4)	0.015
Ischaemic heart disease (IHD)	238 (8.2)	177 (12.8)	0.014
Existing cardiovascular disease	248 (8.5)	188 (10.5)	0.006

*IHD *ischaemic heart disease, *BMI *body mass index, *CV* cardiovascular, *N* number, *SD* standard deviation

*Females

**Males

*** excluding patients who already have cardiovascular diseases

^a^ All P values were calculated using the Chi-square test, except where specifically mentioned

Detailed analysis of wealth index can be downloaded from (http://www.health.gov.lk/moh_final/english/others.php? pid=127).

**Fig. 1:**
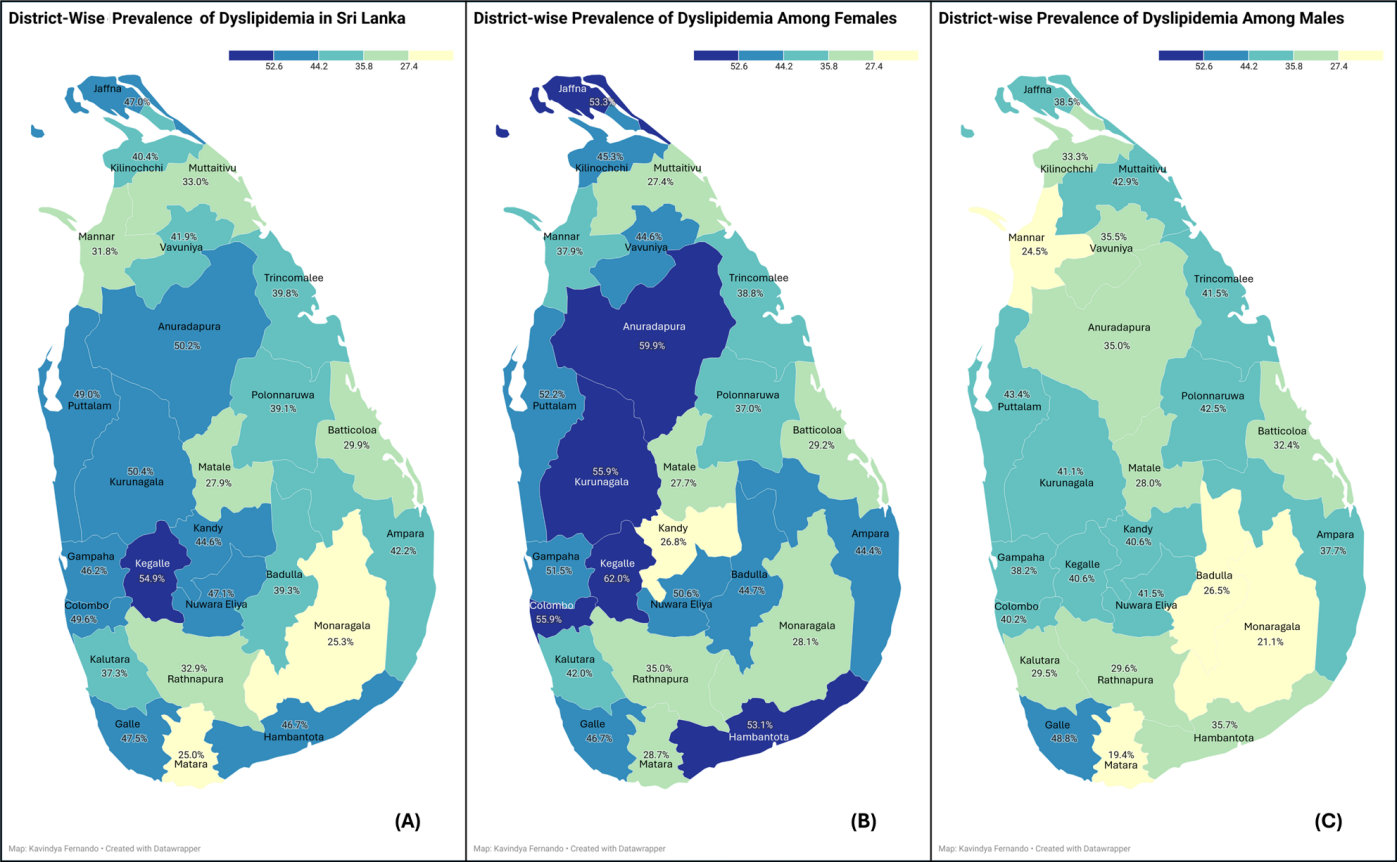
Prevalence rate of hyperlipidaemia in the 25 districts of Sri Lanka. Created via Data wrapper Regional Maps: **A**-Total,**B**- in females, **C**- in males

Figure 1 shows the distribution of hyperlipidaemia by stratified by district and sex.

Table 2 presents the distribution of hyperlipidaemia, treatment uptake and control in the total population and stratified by sex. The overall prevalence of hyperlipidaemia in the sample was 42.6%, with substantial variation across districts of Sri Lanka (Fig. 1; Table 2). The highest prevalence was seen in the Kegalle district, with a prevalence above 52.6%. A higher prevalence of hyperlipidaemia, more than 52.6% was noted in a few districts among females, but not among males. The overall prevalence in the total population was higher in urban (47.0%) and rural areas (42.3%) compared to the estate area (32.6%) (Table 2).

**Table 2:** Prevalence, treatment uptake and control of hyperlipidaemia in the total population and stratified by sex

	Total population^a^ *N*(%) *N* = 4634	Females* N(%) *N* = 2914	Males** N(%) *N* = 1720	*P* value^b^
Had checked cholesterol levels at least once in life, prior to this study	2483 (53.6)	1613 (55.4)	870 (50.6)	0.002
Prevalence of hyperlipidaemia				
In the total population	1975 (42.6)	1349 (46.3)	626 (36.4)	< 0.001
Age < 50 years	976 (36.4)	658(38.0)	318(33.4)	0.017
Age ≥ 50 years	999 (51.2)	691(58.4)	308(40.2)	< 0.001
By the sector of residence n (%)				
Urban (N=*417, **272)	324 (47.0)	219 (52.5)	105 (38.6)	< 0.001
Rural (N=*2396, **1377)	1595 (42.3)	1094 (45.6)	501 (36.3)	< 0.001
Estate (N=*101, **71)	56 (32.6)	36 (35.6)	20 (28.1)	0.194
By the level of education,				0.214
No schooling	49 (2.5)	35 (2.6)	14 (2.2)	
Up to grade 5	295 (14.9)	192 (14.2)	103 (16.5)	
Passed grade 6–10	896 (45.4)	596 (44.2)	300 (47.9)	
Passed G.C.E. O/L	319 (16.2)	232 (17.2)	87 (13.9)	
Passed G.C.E. A/L	336 (17)	239 (17.7)	97 (15.5)	
Degree and above	80 (4.1)	55 (4.1)	25 (4)	
By the Wealth index, n(%)				0.513
Poorest quintile	369 (18.7)	263 (19.5)	106 (16.9)	
Second quintile	396 (20.1)	265 (19.5)	131 (20.9)	
Third quintile	398 (20.2)	276 (20.5)	122 (19.5)	
Fourth quintile	400 (20.3)	274 (20.3)	126 (20.1)	
Richest quintile	412 (20.9)	271 (20.1)	141 (22.5)	
Total cholesterol controlled < 200 mg/dL, n (%)	3015 (65.1)	1796 (61.6)	1219 (70.9)	< 0.001
Treatment of hyperlipidaemia in patients with hyperlipidaemia, n (%) (N = ^a^ 1975, *1349, **626)				
Currently on some treatment	488 (24.7)	340 (25.2)	148(23.6)	0.467
Treatment type				
On a statin	309(63.3)	208(61.2)	101 (68.2)	0.152
On herbal medicine	84 (17.2)	46 (13.5)	38(25.7)	0.002
Other/unspecified	95 (19.5)	86 (25.3)	9 (6.1)	
Source of treatment				0.087
Government hospitals	271 (55.5)	198 (58.2)	73 (49.3)	
Private hospitals	84 (17.2)	57 (16.8)	27 (18.2)	
Pharmacy	19 (3.9)	9 (2.6)	10 (6.8)	
General practitioner	114 (23.4)	76 (22.4)	38 (25.7)	
Total cholesterol goal achievement	321(72.5)	209(68.1)	112(82.4)	0.002

N number, SD standard deviation

^a^ Total population, ^b^ comparison between males and females, *Females, **Males

*P*-values were calculated using the Chi-square test for categorical variables and the independent samples t-test for continuous variables

Detailed analysis of wealth index can be downloaded from (http://www.health.gov.lk/moh_final/english/others.php? pid=127)

Table 2 presents the prevalence of hyperlipidaemia, treatment uptake and TC goal achievement in those treated in the total population and stratified by sex. On average, 2483(53.6%) had checked their lipid levels at least once in their life prior to this study and this rate was significantly higher among females (1613(55.4%)) compared to males (870(50.6%)), *P* = 0.002. Overall, females 1349(46.3%) had a higher prevalence of hyperlipidaemia than males 626(36.4%) (*P* < 0.001). This sex-related difference was significant in both < 50-year-olds (*P* = 0.017) and ≥ 50-year-olds (*P* < 0.001), without showing a major difference in pre-menopausal female group. The sex-related difference was significant in both urban (*P* < 0.001) and rural (*P* < 0.001) sectors but not in the estate sector (*P* = 0.194). The mean number of years of schooling in females with hyperlipidaemia (9.9 years, SD 4.6) was higher than that of males (9.4 years, SD 3.4) (*P* = 0.025) but there was no difference noted in the prevalence of hyperlipidaemia between females and males of different quintiles of wealth index. The mean TC was higher in females (192.8 mg/dL, SD 42.7) than in males (183.8 mg/dL, SD 39.6) (*P* < 0.001). Of all, 3015 (65.1%) of the total population had a TC level below 200 mg/dL in 1796(61.6%) of females vs. 1219(70.9%) of males (*P* < 0.001)).

The rate on treatment for hyperlipidaemia in the total population was 24.7% and was not different between females (25.2%) and males (23.6%), *P* = 0.467 (Table 2). Among all respondents receiving treatment, 309(63.3%) were on a statin, while 84(17.2%) were on herbal medicine. The percentage on statin was not different between females and males (61.2%, 68.2% respectively, *P =* 0.152), but more males (25.7%) used herbal medicine than females (13.5%) (*P* = 0.002). The majority obtained treatment from the state healthcare sector, with no sex-related difference in this pattern. In patients who are on treatment for hyperlipidaemia, the rate of TC goal achievement was significantly lower in females (68.1%) compared to males (82.4%) (*P =* 0.002).

Associations of hyperlipidaemia in the total population and stratified by sex are shown in Table 3. On un-adjusted analyses, age, female sex, level of education, wealth quintile, presence of diabetes mellitus, hypertension, waist circumference, BMI ≥ 23 kg/m^2^ and current smoking all were associated with hyperlipidaemia. However, in mention the method used and the model parameters adjusted analyses hyperlipidaemia was positively associated only with increasing age (OR 1.03, *P* < 0.001), female sex (OR 1.56, *P* < 0.001), increase in wealth quintile (OR 1.09, *P* = 0.002), diabetes mellitus (OR 1.32, *P* = 0.001). When considering associations of hyperlipidaemia in females and males separately, apart from the associations with hyperlipidaemia in the total population, level of education was associated with hyperlipidaemia only in males and not in females. The risk of hyperlipidaemia doubled in females aged 50 years or more compared to females of all ages.

**Table 3:** Factors associated with hyperlipidaemia in females, males and the total population

	Females		Males		Total population				
					LR	LR*			
	OR	*P*	OR	*P*	*P*	aOR*	95% CI	P*	
Age	1.04^a^	< 0.001	1.02 ^a^	< 0.001	< 0.001	1.03	1.022–1.033	< 0.001	
Age ≥ 50 years	2.29	< 0.001	1.34	0.004	< 0.001	…	…	…	
Female sex	…	…	…	…	< 0.001	1.56	1.352–1.802	< 0.001	
Level of education	…	0.299	…	0.002	0.024	0.99	0.932–1.059	0.842	
Wealth quintile	…	0.420	…	0.212	0.021	1.09	1.032–1.142	0.002	
Diabetes mellitus	1.66	< 0.001	1.77	< 0.001	< 0.001	1.32	1.116–1.549	0.001	
Hypertension	1.81	< 0.001	1.18	0.071	< 0.001	1.11	0.956–1.277	0.177	
Waist, cm	1.02 ^a^	< 0.001	1.01 ^a^	< 0.001	< 0.001	1.01	0.999–1.012	0.096	
BMI ≥ 23 kg/m^2^	1.49	< 0.001	1.30	0.010	< 0.001	1.15	0.973–1.35	0.102	
Current smoking	2.16 ^b^	0.099	0.98	0.912 ^b^	0.008	0.95	0.759–1.188	0.649	

LR Logistic regression, OR odds ratio, aOR adjusted odds ratio, CI confidence interval, BMI body mass index.

All calculations for males and females were done using Pearson’s Chi-square unless specifically mentioned as “^b^” which was done using Fisher’s exact test.

^a^ logistic regression.

^b^ Fisher’s exact test.

Logistic regressions were done using binary logistic regression.

* Adjusted for all the variables in the table (age, sex, diabetes mellitus, hypertension present or not, waist circumference, BMI, current smoking, moderate to severe exercise and current alcohol consumption).

**Table:** 

			Hosmer and Lemeshow Test		
−2 Log likelihood	Cox & Snell R Square	Nagelkerke R Square	Chi-square	df	Sig.
5940.721^a^	0.058	0.078	3.49	8	0.9

Of the individuals on treatment for hyperlipidaemia, 209(68.1%) of females and 112 (82.4%) of males achieved TC treatment goals (Table 2). The factors associated with the non-achievement of the TC goal are shown in Table 4. Female sex was significantly associated with non-achievement of TC goal in both adjusted (OR 3.03, *P* < 0.001) and non-adjusted models (OR 2.56, *P* < 0.001). Increasing wealth quintiles showed a marginal association with poor goal achievement in non-adjusted analyses but this association was nullified in adjusted analysis. None of the other factors studied including age, level of education, and metabolic risk factors were associated with the non-achievement of the TC goal.

**Table 4:** Associations of non-achievement of total cholesterol goal in patients treated for hyperlipidaemia (*n* = 443)

	OR	95% CI	*P*-value	aOR*	95% CI	*P*-value
Age	1.00	0.98–1.02	0.973	0.99	0.97–1.02	0.505
Female sex	2.56	1.56–4.22	< 0.001	3.03	1.68–5.47	< 0.001
Level of education	0.92	0.76–1.10	0.356	1.01	0.81–1.26	0.923
Wealth quintile	0.87	0.76–1.00.76.00	0.051	0.94	0.79–1.10	0.424
Diabetes mellitus	0.78	0.51–1.16	0.222	0.78	0.51–1.18	0.233
Hypertension	1.39	0.92–2.07	0.111	1.44	0.94–2.20	0.096
Waist, cm	1.00	0.98–1.01	0.627	0.99	0.97–1.02	0.586
BMI ≥ 23 kg/m^2^	1.08	0.67–1.73	0.755	1.14	0.62–2.07	0.677
Current smoking	1.01	0.41–2.45	0.988	0.41	0.15–1.15	0.089

*LR Logistic regression*,* OR odds ratio*,* aOR adjusted odds ratio*,* CI confidence interval*,* BMI body mass index*

All odds ratios were calculated using binary logistic regression

* Multivariable logistic regression adjusted for age, sex, level of education, wealth quintile, diabetes mellitus, hypertension, waist circumference, BMI and current smoking status

**Table:** 

			Hosmer and Lemeshow Test		
−2 Log likelihood	Cox & Snell R Square	Nagelkerke R Square	Chi-square	df	Sig.
543.934^a^	0.048	0.07	4.747	8	0.784

Of all respondents, 436 (248 (8.5%) females and 188 (10.5%) males) have had pre-existing CVDs. The factors associated with the presence of existing CVDs in the total population and by sex are presented in Table 5. Male sex, older age, presence of comorbidities, namely hyperlipidaemia, diabetes mellitus and hypertension were independent associations of the presence of existing CVDs in the total population. When comparing the associations of existing CVDs stratified by sex, the same factors were associated with existing CVDs in females and males except, increasing waist circumference and overweight being positively associated with existing CVDs only in males (OR 1.02, *P =* 0.016 and OR 1.41, *P* = 0.027, respectively) but not in females (OR 1.01, *P =* 0.314 and OR 1.07, *P =* 0.626). The odds of the association of hyperlipidaemia for CVDs in females (OR 3.23, confidence interval (CI) 2.42–4.33) was higher than that in males (OR 2.79, CI 1.92–4.06). The association of hyperlipidaemia for CVDs in females vs. males was not different in patients less than 50 years and those 50 years or above.

**Table 5:** Factors associated with the presence of existing cardiovascular diseases in females, males and the total population

	Females		Males		In the total population			
					LR	LR *		
	OR	*P*	OR	*P*	*P*	aOR	95% CI	*P*
Age	1.04^a^	< 0.001	1.04 ^a^	< 0.001	< 0.001	1.03	1.02–1.03	< 0.001
Female sex	…	…	…	…	0.009	0.75	0.60–0.95	0.017
Level of education	…	0.036	…	< 0.001	< 0.001	0.8	0.72–0.89	< 0.001
Wealth quintile	…	0.676	…	0.073	0.582	1.07	0.99–1.17	0.109
Hyperlipidaemia	3.23	< 0.001	2.79	< 0.001	< 0.001	2.08	1.61–2.68	< 0.001
In those < 50 years	2.86	< 0.001	2.60	0.010	…	…	…	…
In those ≥ 50 years	2.46	< 0.001	2.25	< 0.001	…	…	…	…
Diabetes mellitus	2.22	< 0.001	1.89	< 0.001	< 0.001	1.37	1.07–1.75	0.012
Hypertension	1.69	< 0.001	2.00	< 0.001	< 0.001	1.23	0.98–1.54	0.076
Waist, cm	1.01	0.314	1.02	0.016	0.009	1.00	0.99–1.01	0.898
BMI ≥ 23 kg/m^2^	1.07	0.626	1.41	0.027	0.160	1.05	0.80–1.39	0.723
Current smoking	1.09^b^	1.000	0.941^b^	0.734	0.108	0.97	0.69–1.37	0.872

*LR Logistic regression*,* OR odds ratio*,* aOR adjusted odds ratio*,* CI confidence interval*,* BMI body mass index*

All calculations for males and females were done using Pearson’s Chi-square unless specifically mentioned

^a^ Logistic regression

^b^ Fisher’s exact test

All logistic regressions were done using binary logistic regression.

* Multivariable logistic regression, including all the variables in the table in the model (age, sex, level of education, Wealth quintile, hyperlipidaemia, diabetes mellitus, hypertension, waist circumference, BMI ≥ 23 kg/m^2^, current smoking status)

**Table:** 

			Hosmer and Lemeshow Test		
−2 Log likelihood	Cox & Snell R Square	Nagelkerke R Square	Chi-square	df	Sig.
2680.217a	0.037	0.08	4.475	8	0.812

Of the total respondents, 438 had existing CVDs. Of the patients who were naïve to CVDs, 2667 of 40–69 years of age had complete data to calculate CV risk predictions. Their 10-year CV risk predictions were calculated using the WHO risk charts. The lifetime CV risk was calculated in the subset of 20–59-year-olds. The association of hyperlipidaemia with high 10-year CV risk (including *10-year CV risk of ≥ 20% or with existing CVDs*) and high *lifetime CV risk (including lifetime risk of ≥ 39% or with existing CVDs*) stratified by sex are shown in Table 6. Of the respondents, 10.9% were at high 10-year CV risk while 39.7% were at high lifetime CV risk (Table 6). Hyperlipidaemia was associated with both high 10-year CV risk as well as high lifetime CV risk. The association of hyperlipidaemia with high 10-year CV risk was stronger in females than in males, both in adjusted and non-adjusted analyses. However, the independent association of hyperlipidaemia with high lifetime CV risk was stronger in males (OR 1.92, 95% CI 1.01–3.67) than in females (OR 1.62, 95% CI 1.00 −2.63) in adjusted analyses.

**Table 6:** Association of hyperlipidemia with cardiovascular risk

	Female				Male					Total population			
	10-year CV risk of ≥ 20% or with existing CVDs 269(9.2%)		Lifetime CV risk of ≥ 39% or with existing CVDs 387(39.2%)		10-year CV risk of ≥ 20% or with existing CVDs 236(13.7%)		Lifetime CV risk of ≥ 39% or with existing CVDs 332(40.2%)			10-year CV risk of ≥ 20% or with existing CVDs 505(10.9%)		Lifetime CV risk of ≥ 39% or with existing CVDs 719(39.7%)	
OR	*P*	OR	*P*	OR	*P*	OR	*P*	OR		*P*	OR	*P*	
Hyperlipidaemia (unadjusted)	3.67	< 0.001	4.56	< 0.001	2.24	< 0.001	4.48	< 0.001	2.29		< 0.001	4.46	< 0.001
Hyperlipidaemia (adjusted*)	2.12	< 0.001	1.62	0.052	1.38	0.124	1.92	0.048	1.74		< 0.001	1.68	0.008

CV cardiovascular, CVD cardiovascular disease

OR odds ratio, P p value

All calculations were done using binary logistic regression

* Multivariable logistic regression adjusted for age, level of education, wealth quintiles, diabetes mellitus, hypertension, waist circumference, body mass index ≥ 23 kg/m2 and current smoking

**Table:** 

Lifetime risk CV risk					Hosmer and Lemeshow Test		
	SexFemale1	−2 Log likelihood	Cox & Snell R Square	Nagelkerke R Square	Chi-square	df	Sig.
	Male	662.873^a^	0.418	0.566	20.547	8	0.008
	Female	853.927^b^	0.375	0.508	22.399	8	0.004
	Total sample	1531.438^a^	0.39	0.528	21.447	8	0.006
10-year CV risk					Hosmer and Lemeshow Test		
	SexFemale1	−2 Log likelihood	Cox & Snell R Square	Nagelkerke R Square	Chi-square	df	Sig.
	Male	1185.995^a^	0.099	0.179	21.991	8	0.005
	Female	1620.875^b^	0.051	0.109	6.372	8	0.606
	Total sample	2825.653^a^	0.069	0.139	26.439	8	< 0.001

## Discussion

Sri Lankan females, notably those aged above 50 years, demonstrated a higher prevalence of hyperlipidaemia and poor TC goal achievement compared to males despite similar treatment uptake rates, even when adjusted for other confounders like the level of education, wealth index, and other metabolic risk factors. In contrast, the prevalence of CVDs was higher among males. Male sex, age, hyperlipidaemia, diabetes mellitus and hypertension were independent associations of the presence of atherosclerotic CVDs. Even though female sex was negatively associated with CVDs, hyperlipidaemia showed a stronger association with existing CVDs in females than in males in stratified analysis. The independent association of hyperlipidaemia with high 10-year CV risk was higher in females compared to males. However, the independent association of hyperlipidaemia with lifetime CV risk was higher in males compared to women.

The findings of this study align with and expand upon observations from other South Asian and international studies. A decade ago, the Sri Lanka Diabetes and Cardiovascular Study (2005–2006) similarly reported that females had higher mean TC, LDL-C, HDL-C, and triglyceride levels than males across all age groups [26]. Notably, that study identified female sex as an independent risk factor for hyperlipidaemia, along with age and adiposity [26] ​. Studies from other Asian countries have reported comparable patterns; for example, an extensive Chinese study noted that middle-aged females had a higher prevalence of borderline-high or elevated TC, LDL-C, and triglycerides than males, especially beyond the fourth decade of life [39]​.

Globally, it is recognised that females tend to have higher TC and HDL-C levels than males throughout much of adult life [22]. This ‘female cholesterol excess’ has been partly attributed to hormonal influences – oestrogen raises HDL-C and can delay the rise in LDL-C until after menopause [2]. By midlife, as ovarian hormone levels decline, females often experience surges in LDL-C and triglycerides, surpassing levels observed in males [2].

The higher mean BMI and overweight rates observed in females in this study (64.2% with BMI ≥ 23 kg/m² vs. 50.2% of males) likely contribute significantly to their more adverse lipid profiles. South Asian females, especially in urban settings, may have lower physical activity levels and dietary patterns predisposing to weight gain and hyperlipidaemia due to sociocultural norms (e.g. sedentary domestic roles, dietary practices post-pregnancy) [40]. All these factors may cumulatively result in greater lifetime exposure to atherogenic lipids in females [2]. It is worth noting, however, that not all studies in Sri Lanka have found sex-related differences – a recent community survey in one province, for instance, did not identify sex as a significant determinant of hyperlipidaemia​ [27]. That null finding could reflect a smaller, younger sample or regional lifestyle factors. In contrast, current nationally representative data and previous large-scale surveys suggest a consistent sex-related difference in lipid profiles.

Treatment uptake was similar in females and males, in contrast to findings reported in previous literature [15, 41, 42]. Although statin use did not differ by sex in the present sample, females were less likely than males to use herbal therapies for lipid-lowering. This observation differs from the trends in many high-income settings, where females have historically been less likely than males to be prescribed statins or to receive intensive lipid-lowering therapy [14, 43]. For instance, a managed-care analysis in the United States noted that females were less often screened, treated, or at LDL-C goal than males [14] ​. This difference may be explained by the high literacy rate in both females (94.3%) and males (92.3%) and less sex-related inequality in Sri Lanka compared to most Asian countries [44–46].

Nevertheless, the lower control rate in females points to gaps in the quality and intensity of treatment in addition to the effect of biological sex. Potential factors include suboptimal dosing or adherence: prior research indicates females are more likely to discontinue statin therapy than males, even when eligible [14]. They also may be less likely to be escalated to high-intensity statins due to concerns about side effects or a misperception that their risk is lower [14]. As a result, females often do not reach lipid targets. For example, a recent registry from a tertiary centre showed only 30% of females with established CVDs achieved LDL-C < 70 mg/dL, compared to 43% of males [43]​. This study mirrors this pattern of poorer goal attainment in females despite their greater uptake of therapy. This highlights an urgent need to improve not just access to therapy but also adherence and treatment optimisation in females. Culturally sensitive education about medication importance, managing statin side effects, and clinician vigilance in up-titrating doses for females are warranted to close the control gap [43]​ ​.

A familiar paradox was noted: females have more hyperlipidaemia yet lower CV event rates, which cannot be explained simply by sex. We noted that the association of hyperlipidaemia and 10-year CV risk was stronger in females but the association with lifetime CV risk was less strong in females compared to men. It is well known that females experience major atherosclerotic events approximately 5–10 years later than males on average [44]. Multiple biological and sociocultural factors likely underlie the observed gender disparities. Beyond the effects of endogenous hormones on lipids, females face unique risk modifiers: conditions such as polycystic ovary syndrome, hypertensive disorders of pregnancy (e.g. preeclampsia), gestational diabetes, and early menopause, which are all associated with hyperlipidaemia and CVDs [2]. These factors are not captured in conventional risk scores but could contribute to an elevated risk trajectory in females that becomes apparent later in life. Females in this study had a greater BMI and obesity, which is often linked to a higher cardiometabolic burden. South Asian females may have more adverse fat distribution and inflammatory profiles at a given BMI than males [47, 48].

Sociocultural components are undoubtedly at play [2] ​. In many South Asian communities, men traditionally engage in more physical activities (through labour or leisure), whereas women may be less active due to household responsibilities and social norms limiting exercise [47]. Dietary differences, possibly influenced by cultural practices (such as women eating last or consuming carbohydrate-rich staples), might also affect lipid levels [49–51]. Psychosocial stressors and mental health, including depression and anxiety, are reported to be more prevalent in females and can indirectly worsen CV risk by hindering healthy behaviours​ [10, 19]. Moreover, awareness of CV risk is often lower in females; historically, heart disease has been perceived as a “male” problem, leading to females being less likely to recognise symptoms or seek preventive care [44]​. Low health literacy and socioeconomic disadvantages among females can further exacerbate this. Notably, a large meta-analysis found that low socioeconomic status confers a greater relative risk of CVDs in females than in males [19]. Females of lower socioeconomic strata may have less access to care, poorer nutrition, and higher exposure to chronic stress, amplifying their CV risk.

Overall, these data reinforce that females have a lower CV risk, especially pre-menopausal, irrespective of their lipid levels, but are not protected from CVDs as their risk manifests later in life. Global trends are concerning in this regard: in the last decade, CV mortality rates have been rising in females, with middle-aged females showing the fastest relative increase in atherosclerotic CVDs. The findings of this study caution that a woman with multiple risk factors (e.g. high cholesterol, obesity, hypertension) should not be deemed low-risk simply because her 10-year risk score is below a treatment threshold. Short-term risk calculators can underestimate risk in females​ [44] who often accumulate risk factor exposure over an extended period before an event occurs. Thus, a more nuanced approach to risk assessment in females is needed, considering lifetime risk and the presence of risk enhancers unique to females.

### Strengths and limitations

There are several strengths in this study. This is a nationally representative sample of Sri Lanka, and the methodology was robust, hence giving the best possible picture of sex and hyperlipidaemia.

However, there are a few limitations to acknowledge. The analysis was limited to individuals with TC measurements available, which may have excluded those less likely to undergo lipid testing or participate in the survey, potentially introducing selection bias. CV risk predictions were calculated only for individuals with complete data, which may have introduced a similar selection bias. However, this bias is likely minimal due to a multi-stage stratified cluster sampling method. Only TC was assessed due to financial constraints. Including additional lipid parameters would have provided a more comprehensive evaluation of the population’s CV risk and lipid goal achievement. We used the ASCVD lifetime risk tool to calculate lifetime CV risk in 20–59-year-olds without existing CVDs, as there are no lifetime risk calculators specific for Sri Lankans or South Asians. These estimates may underestimate/overestimate lifetime risk for Asians. However, the effect of this on gender disparity is minimal, as we compared the lifetime risk of females and males using the same tool.

Medication-related data, like medication adherence and intensity of prescribed statins, were unavailable to comment on, as the original STEPS survey data set did not have complete data on these parameters. We collected data of self-reported CVDs and therefore, this may have introduced some recall and misclassification bias. However, this bias was present for both sexes and therefore the chance of this bias affecting final conclusions would be minimal. Further, confounding from unmeasured female-specific risk enhancers like early menopause, previous pregnancy-related disorders or hormonal therapy was not studied due to the unavailability of data. Self-reported CVD history may introduce bias. However, our stratified random sampling method captures the effect/outcome of those risk enhancers on the prevalence of hyperlipidaemia. The identified sex related disparities between sexes could have been explained well had this data been available.

Data on existing CVDs were based on the self-reported history from patients. This could have introduced some recall bias.

## Conclusion

Sri Lankan females had a higher prevalence and poorer control of hyperlipidaemia compared to males, despite no difference in treatment uptake or when adjusted for other confounders like the level of education, wealth index, and other metabolic risk factors. Even though the presence of CVDs was higher among males, it was independently associated with sex and hyperlipidaemia. Therefore, there is a critical need for the medical community to make a paradigm change in the prevention of CVDs in females. Routine screening for lipid disorders and other CV risk factors should be strengthened in females and males, especially at younger ages and during key healthcare encounters. When hyperlipidaemia is identified, prompt initiation of therapy and meticulous follow-up to ensure control are essential – this may involve patient education, overcoming therapeutic inertia, and addressing sex-related barriers to adherence. Tailored public health measures for females are needed: lifestyle modification programs that are culturally appropriate and accessible, efforts to improve females’ knowledge of nutrition and physical activity, and community campaigns to raise awareness. A comprehensive approach, combining early risk detection, equitable treatment, and the empowerment of females to adopt heart-healthy lifestyles, is essential to address the rising burden of CVDs in South Asian females. Improving the knowledge of females on the importance of controlling lipids and the necessary measures, especially breaking cultural barriers in females engaging in physical activity, takes priority, as more females have poorly controlled hyperlipidaemia compared to men, but with similar treatment uptake.

## Supplementary Information

Supplementary material 1.

## Data Availability

The datasets used and analysed during this study are available from the corresponding author upon reasonable request.

## Abbreviations

ASCVD: Atherosclerosis-related cardiovascular disease
BMI: Body mass index
CI: Confidence interval
CV: Cardiovascular
CVD: Cardiovascular disease
FBS: Fasting blood sugar
HDL-C: High-density lipoprotein cholesterol
IHD: Ischaemic heart disease
IQR: Interquartile range
LDL-C: Low-density lipoprotein cholesterol
LR: Logistic regression
NCD: Noncommunicable disease
NCEP: National cholesterol education programme
OR: Odds ratio
aOR: Adjusted odds ratio
TC: Total cholesterol
TG: Triglycerides
SBP: Systolic blood pressure
SD: Standard deviation
WHO: World health organization

## References

[1] Di Cesare M, et al. The heart of the world. Glob Heart. 2024. 10.5334/gh.1288.39479259

[2] van Roeters Lennep JE, et al. Women, lipids, and atherosclerotic cardiovascular disease: a call to action from the European Atherosclerosis Society. Eur Heart J. 2023;44(39):4157–73. 37611089 10.1093/eurheartj/ehad472 PMC10576616

[3] Ndzie Noah ML, et al. Sex-gender disparities in cardiovascular diseases: the effects of Estrogen on eNOS, lipid profile, and NFATs during catecholamine stress. Front Cardiovasc Med. 2021;8:639946. 33644139 10.3389/fcvm.2021.639946 PMC7907444

[4] Mahowald MK, et al. Sex disparities in cardiovascular disease. Methodist Debakey Cardiovasc J. 2024;20(2):107–19. 38495656 10.14797/mdcvj.1328 PMC10941692

[5] Elder P, et al. Identification of female-specific risk enhancers throughout the lifespan of women to improve cardiovascular disease prevention. Am J Prev Cardiol. 2020;2:100028. 34327455 10.1016/j.ajpc.2020.100028 PMC8315406

[6] Motairek I, et al. Sex differences in cardiovascular mortality among patients with immune mediated inflammatory diseases. Circ Cardiovasc Qual Outcomes. 2025;18(5):e011833. 40321135 10.1161/CIRCOUTCOMES.124.011833

[7] Moran CA, et al. Cardiovascular implications of immune disorders in women. Circ Res. 2022;130(4):593–610. 35175848 10.1161/CIRCRESAHA.121.319877 PMC8869407

[8] Kreatsoulas C, Anand SS. The impact of social determinants on cardiovascular disease. Can J Cardiol, 2010. 26 Suppl C(Suppl C): p. c8–13. 10.1016/s0828-282x(10)71075-8 PMC2949987 20847985

[9] Rajendran A, et al. Sex-specific differences in cardiovascular risk factors and implications for cardiovascular disease prevention in women. Atherosclerosis. 2023;384:117269. 37752027 10.1016/j.atherosclerosis.2023.117269 PMC10841060

[10] Connelly PJ, et al. The importance of gender to understand sex differences in cardiovascular disease. Can J Cardiol. 2021;37(5):699–710. 33592281 10.1016/j.cjca.2021.02.005

[11] Karalis DG, et al. Gender differences in side effects and attitudes regarding statin use in the Understanding Statin Use in America and Gaps in Patient Education (USAGE) study. J Clin Lipidol. 2016;10(4):833–41. 27578114 10.1016/j.jacl.2016.02.016

[12] Gheorghe G, et al. Cardiovascular risk and statin therapy considerations in women. Diagnostics. 2020. 10.3390/diagnostics10070483. 32708558 10.3390/diagnostics10070483 PMC7400394

[13] Bhardwaj S, Selvarajah S, Schneider EB. Muscular effects of statins in the elderly female: a review. Clin Interv Aging. 2013;8:47–59. 23355775 10.2147/CIA.S29686 PMC3552608

[14] Rodriguez F, et al. Gender disparities in lipid-lowering therapy in cardiovascular disease: insights from a managed care population. J Womens Health. 2016;25(7):697–706. 10.1089/jwh.2015.5282 26889924

[15] Zhang X, et al. Gender disparities in lipid goal attainment among type 2 diabetes outpatients with coronary heart disease: results from the CCMR-3B study. Sci Rep. 2017;7(1):12648. 28978912 10.1038/s41598-017-13066-z PMC5627285

[16] Ebong IA, et al. The role of psychosocial stress on cardiovascular disease in women: JACC State-of-the-Art review. J Am Coll Cardiol. 2024;84(3):298–314. 38986672 10.1016/j.jacc.2024.05.016 PMC11328148

[17] Alshakarah A, et al. Awareness and knowledge of cardiovascular diseases and its risk factors among women of reproductive age: a scoping review. Cureus. 2023;15(12):e49839. 38164316 10.7759/cureus.49839 PMC10758256

[18] Simpson M. Women’s heart health: awareness to action. Br J Card Nurs. 2022;17(4):1–3.38812658

[19] Backholer K, et al. Sex differences in the relationship between socioeconomic status and cardiovascular disease: a systematic review and meta-analysis. J Epidemiol Community Health. 2017;71(6):550–7. 27974445 10.1136/jech-2016-207890

[20] Mathews L, et al. Psychological factors and their association with ideal cardiovascular health among women and men. J Womens Health. 2018;27(5):709–15. 10.1089/jwh.2017.6563 PMC5962331 29377738

[21] Vervoort D, et al. Addressing the global burden of cardiovascular disease in women: JACC State-of-the-Art review. J Am Coll Cardiol. 2024;83(25):2690–707. 38897679 10.1016/j.jacc.2024.04.028

[22] Holven KB, van Roeters Lennep J. Sex differences in lipids: a life course approach. Atherosclerosis. 2023;384:117270. 37730457 10.1016/j.atherosclerosis.2023.117270

[23] Kolovou GD, et al. Gender differences in the lipid profile of dyslipidemic subjects. Eur J Intern Med. 2009;20(2):145–51. 19327602 10.1016/j.ejim.2008.06.011

[24] Snieder H, van Doornen LJP, Boomsma DI. Dissecting the Genetic Architecture of Lipids, Lipoproteins, and Apolipoproteins. Arterioscler Thromb Vasc Biol. 1999;19(12):2826–34. 10591657 10.1161/01.atv.19.12.2826

[25] Makshood M, Post WS, Kanaya AM. Lipids in South Asians: epidemiology and management. Curr Cardiovasc Risk Rep. 2019. 10.1007/s12170-019-0618-9. 33833849 10.1007/s12170-019-0618-9 PMC8026164

[26] Katulanda P, et al. Prevalence, patterns, and associations of dyslipidemia among Sri Lankan adults—Sri Lanka Diabetes and Cardiovascular Study in 2005–2006. J Clin Lipidol. 2018;12(2):447–54. 29429894 10.1016/j.jacl.2018.01.006

[27] Nandasena HMRKG, Tennakoon TMSUB, Ralapanawa DMPUK. Prevalence and determinants of dyslipidemia among adults in the community: a cross-sectional study in a selected province, Sri Lanka. Clin Epidemiol Glob Health. 2023;24:101442.

[28] Sri Lanka STEPS Noncommunicable Disease Risk Factors Survey. 2021. 2021; Available from: https://ghdx.healthdata.org/record/sri-lanka-steps-noncommunicable-disease-risk-factors-survey-2021

[29] Non. Communicable diseases risk factor survey (STEPS survey) Sri Lanka 2021, Colombo 14: Sumathi Printers (Pvt) Ltd.

[30] Third Report of the National Cholesterol Education Program (NCEP). Expert panel on Detection, Evaluation, and treatment of high blood cholesterol in adults (Adult treatment panel III) final report. Circulation. 2002;106(25):3143–421. 12485966

[31] Heidari S, et al. Sex and gender equity in research: rationale for the SAGER guidelines and recommended use. Res Integr Peer Rev. 2016;1(1):2. 29451543 10.1186/s41073-016-0007-6 PMC5793986

[32] Collins D, et al. WhoishRisk ? An R package to calculate WHO/ISH cardiovascular risk scores for all epidemiological subregions of the world [version 2; peer review: 3 approved]. Volume 5. F1000Research; 2017. 2522. 10.12688/f1000research.9742.1 PMC5345772 28357040

[33] Kaptoge S, et al. World health organization cardiovascular disease risk charts: revised models to estimate risk in 21 global regions. Lancet Global Health. 2019;7(10):e1332–45. 31488387 10.1016/S2214-109X(19)30318-3 PMC7025029

[34] WHO, World health organization cardiovascular disease risk charts: revised models to estimate risk in 21 global regions. Lancet Glob Health, 2019. 7(10): pp. e1332–45. 10.1016/S2214-109X(19)30318-3 PMC7025029 31488387

[35] Lloyd-Jones DM, et al. Use of risk assessment tools to guide decision-making in the primary prevention of atherosclerotic cardiovascular disease: a special report from the American Heart Association and American College of Cardiology. Circulation. 2019;139(25):e1162-77. 30586766 10.1161/CIR.0000000000000638

[36] Goff DC Jr., et al. 2013 ACC/AHA guideline on the assessment of cardiovascular risk: a report of the American college of Cardiology/American heart association task force on practice guidelines. Circulation. 2014;129(25 Suppl 2):S49–73. 24222018 10.1161/01.cir.0000437741.48606.98

[37] Silva P, Moonesinghe L. Menopause in Sri Lankan context. Sri Lanka Journal of Menopause. 2020. 10.4038/sljom.v2i1.36.

[38] Jayasekara R. Factors influencing the age at natural menopause in sinhalese women. Ceylon J Med Sci. p. 1994;37:2.

[39] Wang M, et al. Gender heterogeneity in dyslipidemia prevalence, trends with age and associated factors in middle age rural Chinese. Lipids Health Dis. 2020;19(1):135. 32532299 10.1186/s12944-020-01313-8 PMC7291723

[40] Waidyatilaka I, et al. Sedentary behaviour and physical activity in South Asian women: time to review current recommendations? PLoS One. 2013;8(3):e58328. 23472180 10.1371/journal.pone.0058328 PMC3589267

[41] Visseren FLJ, et al. 2021 ESC guidelines on cardiovascular disease prevention in clinical practice. Eur Heart J. 2021;42(34):3227–337. 34458905 10.1093/eurheartj/ehab484

[42] van Oortmerssen JAE, et al. Lipid lowering therapy utilization and lipid goal attainment in women. Curr Atheroscler Rep. 2025;27(1):29. 39873822 10.1007/s11883-025-01275-1 PMC11775078

[43] Shahid I, et al. Gender disparities in utilization of Statins for low density lipoprotein management across the spectrum of atherosclerotic cardiovascular disease: insights from the Houston Methodist cardiovascular disease learning health system registry. Am J Prev Cardiol. 2024;19:100722. 39281350 10.1016/j.ajpc.2024.100722 PMC11402022

[44] Sallam T, Watson KE. Predictors of cardiovascular risk in women. Womens Health Lond. 2013;9(5):491–8. 24007254 10.2217/whe.13.44 PMC6097244

[45] UNFPA, Rapid Gender Analysis Sri Lanka 2022. 2023.

[46] Asian D. Bank, Country gender assessment: Sri Lanka. 2008.

[47] Ranasinghe CD, et al. Physical activity patterns among South-Asian adults: a systematic review. Int J Behav Nutr Phys Act. 2013;10(1):116. 24119682 10.1186/1479-5868-10-116 PMC3854453

[48] Santorelli G et al. Differences in total and regional body fat and their association with BMI in UK-born white and South Asian children: findings from the born in Bradford birth cohort [version 1; peer review: 1 approved with reservations]. Wellcome Open Res, 2021. 6(65). 10.12688/wellcomeopenres.16659.3 PMC10611948 37900936

[49] Weerasekara PC, et al. Understanding dietary diversity, dietary practices and changes in food patterns in marginalised societies in Sri Lanka. Foods. 2020;9(11):1659. 33202762 10.3390/foods9111659 PMC7696452

[50] Jayawardena R, et al. High dietary diversity is associated with obesity in Sri Lankan adults: an evaluation of three dietary scores. BMC Public Health. 2013;13(1):314. 23566236 10.1186/1471-2458-13-314 PMC3626879

[51] Renzella J, et al. Food labour, consumption hierarchies, and diet decision-making in Sri Lankan households: a qualitative study. BMC Nutr. 2020;6(1):64. 33292762 10.1186/s40795-020-00389-w PMC7678094

