# Supplementary material for: Sex-related disparities in hyperlipidaemia and cardiovascular risk in Sri Lanka: a cross-sectional study
Source: Lipids Health Dis. 2025 Dec 9;25:13. doi: 10.1186/s12944-025-02812-2 (PMC12802009; doi:10.1186/s12944-025-02812-2)
Supplement: Supplementary file 1 — Supplementary material 1. [file 12944_2025_2812_MOESM1_ESM.doc]

Supplementary Table 1

|  | Urban | Rural | Estate | Total |
| --- | --- | --- | --- | --- |
| Serum Cholesterol tested, n(%) | 698 (71.4) | 3832 (76.2) | 173 (66.5) | 4703 (75.0) |
| Serum Cholesterol not tested, n(%) | 279 (28.6) | 1198 (23.8) | 87 (33.5) | 1564 (25.0) |
| Total | 977 | 5030 | 260 | 6267 |
